# Key bottlenecks to the provision of safe obstetric anaesthesia in low- income countries; a cross-sectional survey of 64 hospitals in Uganda

**DOI:** 10.1186/s12884-017-1566-3

**Published:** 2017-11-17

**Authors:** Isabella Epiu, Agnes Wabule, Andrew Kambugu, Harriet Mayanja-Kizza, Jossy Verel Bahe Tindimwebwa, Gerald Dubowitz

**Affiliations:** 1 Global Health Fellow UCGHI, San Francisco, CA, USA; 2 Makerere University College of Health Sciences, Kampala, Uganda; 3 Infectious Disease Institute, Kampala, Uganda; 4 University of California, San Francisco (UCSF), San Francisco, CA USA; 5 Kenyatta University School of Medicine, P.O.BOX 43844 00100, Nairobi, Kenya

**Keywords:** Safe Anaesthesia, Obstetric Anaesthesia, Low-income countries, Caesarean section, Health system, Maternal mortality, Quality, Universal health care

## Abstract

**Background:**

Despite recent advances in surgery and anaesthesia which significantly improve safety, many health facilities in low-and middle-income countries (LMICs) remain chronically under-resourced with inability to cope effectively with serious obstetric complications (Knight et al., PLoS One 8:e63846, 2013). As a result many of these countries still have unacceptably high maternal and neonatal mortality rates. Recent data at the national referral hospitals in East Africa reported that none of the national referral hospitals met the World Federation of Societies of Anesthesiologists (WFSA) international standards required to provide safe obstetric anaesthesia (Epiu I: Challenges of Anesthesia in Low-and Middle-Income Countries. WFSA; 2014 http://wfsa.newsweaver.com/Newsletter/p8c8ta4ri7a1wsacct9y3u?a=2&p=47730565&t=27996496). In spite of this evidence, factors contributing to maternal mortality related to anaesthesia in LMICs and the magnitude of these issues have not been comprehensively studied. We therefore set out to assess regional referral, district, private for profit and private not-for profit hospitals in Uganda.

**Methods:**

We conducted a cross-sectional survey at 64 government and private hospitals in Uganda using pre-set questionnaires to the anaesthetists and hospital directors. Access to the minimum requirements for safe obstetric anaesthesia according to WFSA guidelines were also checked using a checklist for operating and recovery rooms.

**Results:**

Response rate was 100% following personal interviews of anaesthetists, and hospital directors. Only 3 of the 64 (5%) of the hospitals had all requirements available to meet the WFSA International guidelines for safe anaesthesia. Additionally, 54/64 (84%) did not have a trained physician anaesthetist and 5/64 (8%) had no trained providers for anaesthesia at all. Frequent shortages of drugs were reported for regional/neuroaxial anaesthesia, and other essential drugs were often lacking such as antacids and antihypertensives. We noted that many of the anaesthesia machines present were obsolete models without functional safety alarms and/or mechanical ventilators. Continuous ECG was only available in 3/64 (5%) of hospitals.

**Conclusion:**

We conclude that there is a significant lack of essential equipment for the delivery of safe anaesthesia across this region. This is compounded by the shortage of trained providers and inadequate supervision. It is therefore essential to strengthen anaesthesia services by addressing these specific deficiencies. This will include improved training of associate clinicians, training more physician anaesthetists and providing the basic equipment required to provide safe and effective care. These services are key components of comprehensive emergency obstetric care and anaesthetists are crucial in managing critically ill mothers and ensuring good surgical outcomes.

## Background

Low- and middle-income countries are faced with many competing challenges and public health threats, which are often exacerbated by poor health care. Global initiatives to improve public health have traditionally focused on immunization, sanitation, nutrition, and infectious diseases in low and middle income countries (LMICs). The total volume of surgery undertaken worldwide has recently been estimated for 2004 as 234 million (95% confidence interval, 187.2 million-281.2 million) procedures per year, in comparison with 136 million births worldwide in 2006 [1]. Obstetrics is therefore critical to public health, with safe surgery and safe anaesthesia being critical components of comprehensive emergency obstetric care.

Anaesthesia is one of the medical specialties which has sought to improve the quality of health care and patient safety in these regions by setting standards for safe practice in anesthesia that were adopted by the World Federation of Societies of Anesthesiologists (WFSA) [2]. These standards were specifically determined to ensure minimal error and prevent harm to patients by addressing the needs of LMICs whereby the presence of an appropriately trained and experienced anesthetist is the main determinant of patient safety during anesthesia. These standards also include a provision for monitoring, which has been shown to reduce risks by detecting the consequences of errors as well as giving early warning when the condition of a patient deteriorates [3, 4]. Therefore it is expected that high-level facilities offering anesthesia during major operations, should be meeting the minimum WFSA standards. However, evidence from a recent study conducted at five national referral hospitals across East Africa (Uganda, Kenya, Tanzania, Rwanda and Burundi) showed that none of these hospitals had all the WFSA requirements available to provide safe obstetric anaesthesia [5]. Some of the documented challenges anaesthetists and surgeons face in many low-income countries include a critical shortage of trained providers, poor facilities and a lack of essential drugs, equipment and supplies [6]. Additionally, there is limited published information describing the standards of anaesthesia practice not only at national referral hospitals but also at regional and district hospitals as these provide most anaesthetic care in such countries. Specifically caesarian sections (CS) are top priority of the World Health Organization (WHO) [7]. We therefore set out to estimate the proportion of high level hospitals in Uganda that meet the WFSA international standards, specifically among those that provide obstetric anaesthesia. Additionally, we sought to identify key bottlenecks in the provision of safe obstetric anaesthesia. This is important to improve patient safety by implementing recommendations to minimize the risks and addressing some of the main causes that lead to poor outcomes [8].

## Methods

This was a cross-sectional survey conducted in Uganda from September 2014 to August 2015. A total of 64 hospitals across Uganda were selected based on the criteria that they provided obstetric anaesthesia. At least 15 hospitals from each region; East, West, North and Central were included for representativeness. This study was part of a larger comprehensive survey of the emergency and anaesthesia services in Uganda conducted during the corresponding authors’ National Institutes of Health (NIH) funded fellowship in Global Health where peri-operative data was collected following the World Federation of Societies’ of Anaesthesiologists (WFSA) international guidelines for safe anaesthesia. A survey tool to evaluate compliance was developed based on WFSA Guidelines and the WHO Safe surgery checklist. Additionally we evaluated demographic data on staffing, availability of equipment, monitors, and drugs. These included pre-operative assessment of patients, staffing and continuous monitoring intra-operatively and post-operatively. In this report we have included the peri-operative components of staffing, availability of equipment, monitors, and drugs recommended for safe anaesthesia by the WFSA.

We purposefully selected all the 12 regional referral hospitals because these are level 3 centres and the lower health centres usually refer patients here for surgery. We also randomly selected hospitals from the other groups to include general (government district hospitals), private for profit and private not for profit hospitals.

The survey tool comprised of 3 components, the first was an interviewer-administered questionnaire to one Anaesthetist available at each hospital with the aim of understanding the quality of anaesthesia care. The second was a checklist to objectively assess the obstetric theatres. The third was another interviewer-administered questionnaire with the Directors of the hospital in order to document other challenges faced in delivery of anaesthesia care at the government and private hospitals. Data on caesarean sections and anaesthetists countrywide was obtained from the ministry of health.

With the help of the statistician, data was subsequently cleaned and, coded, into Epidata version 3.1. Range, consistency and validity checks were built in to minimize errors. Data was exported and analyzed using STATA version 14 (Statcorp, College Station, Texas, USA). We dichotomised according to drugs and 15 facility variables available in theatre and postoperative recovery areas including functional anaesthesia machine, oxygen source, reservoir oxygen source, continuous Blood Pressure, Continuous ECG, and Continuous pulse oximetry, suction machine, laryngoscope, Endotracheal tubes (ETT), Face Masks and Laryngeal Mask Airways (LMA), Stethoscope, Difficult Airway Cart, Defibrillator, Capnograph and availability of ICU facilities for post-operative care of complicated cases.

Ethical approval was obtained from Makerere University School of Medicine Research and Ethics Committee (SOMREC), the appropriate hospital ethics committees for participating hospitals, and the Uganda National Council for Science and Technology Ethics Committee. Informed consent was obtained from all individuals participating in the study.

## Results

**Fig. 1:**
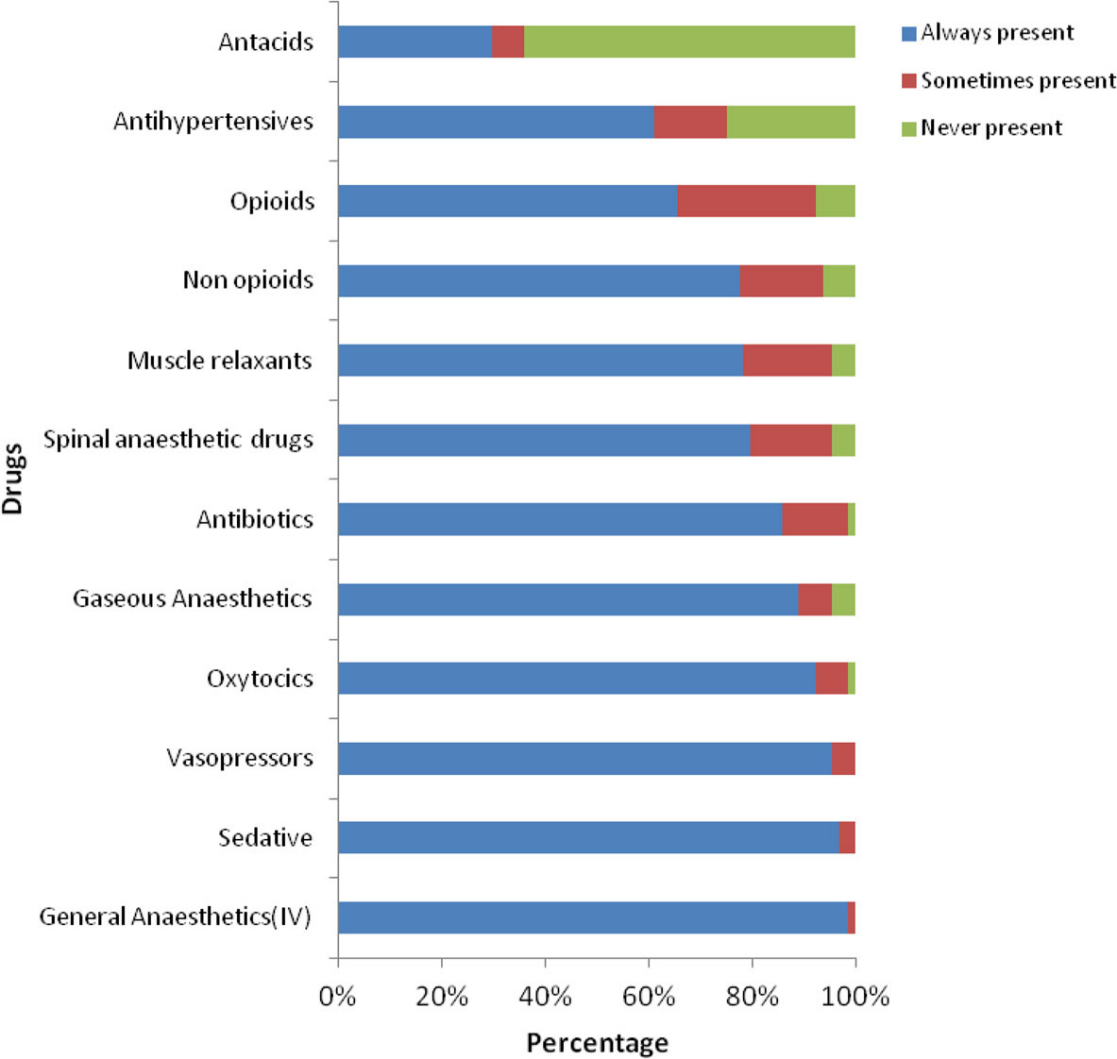
Availability of drugs for anaesthesia in Hospitals in Uganda

We contacted 64 hospitals in Uganda, 41% of all the hospitals in the country [9]. The hospital directors reported the major challenges faced during delivery of obstetric anaesthesia being few anaesthesia providers. Of 64 hospitals evaluated, 54/65 (84%) did not have a trained physician anaesthetist, 59/64 (92.2%) had ≥1 nurse anaesthetist and 5/64 (8%) had no trained providers for anaesthesia (Table 1). There were reports of deficiencies in regional anaesthetics supplies throughout the country, and other essential drugs like antacids and anti-hypertensive drugs (Fig. 1). The availability of monitors for anaesthesia varied (Table 2).

**Table 1:** Baseline characteristics of anaesthetists interviewed

Characteristic	Distribution of hospital regional locations				
	*N*(%)				
	Central	East	West	North	Overall
	*N* = 15	*N* = 18	N = 15	*N* = 16	*N* = 64
Mean age in years (SD)^a^	44.09(10.8)	47.69(10.3)	39.87(6.7)	46.06(8.7)	44.53(9.4)
Mean years of experience (SD)^b^	13.43(8.7)	16.67(12.3)	9.20(5.3)	14.06(10.5)	13.51(9.9)
Sex					
Female	8(53.3)	1(77.8)	10(66.7)	11(68.8)	43(67.2)
Male	7(46.7)	4(22.2)	5(33.3)	5(31.3)	21(32.8)
Anaesthetist level of training					
Physician	2(13.3)	0	2(13.3)	1(6.3)	5(7.8)
Nurse anesthetist	13(86.7)	16(88.9)	11(73.3)	12(75.00)	52(81.3)
Clinical officer	0	0	2(13.3)	1(6.3)	3(4.7)
Other	0	2(11.1)	0	2(12.5)	4(6.3)
Another place of work					
Private	5(33.3)	14(77.8)	10(66.7)	12(75.0)	41(64.1)
None	10(66.7)	4(22.2)	5(33.3)	4(25.0)	23(35.9)
Presence of 24 h recovery room					
No	4(26.7)	16(88.9)	8(53.3)	12(75.0)	40(62.5)
Yes	11(73.3)	2(11.1)	7(46.7)	4(25.0)	24(37.5)
Number of physician anaesthetists working at the hospital					
None	10(66.7)	17(94.4)	13(86.7)	14(87.5)	54(84.4)
≥ 1	5(33.3)	1(5.6)	2(13.3)	2(12.5)	10(15.6)
Number of nurse anaesthetists working at the hospital					
None	0	2(11.1)	0	3(18.8)	5(7.8)
≥ 1	15(100)	16(88.9)	15(100)	13(81.3)	59(92.2)

^a^Missing 6 age entries

^b^Missing 1 years of experience entry

**Table 2:** Distribution of instruments present by hospital category

	Overall	Regional referral hospitals	General hospitals	Private for profit hospitals	Private Not for Profit Hospitals
	*N* = 64	*N* = 13	*N* = 21	*N* = 7	*N* = 23
Anaesthetic machine					
Reserve oxygen cylinder	29(45.3)	8(61.5)	7(35.0)	5(83.3)	9(39.1)
Inspired oxygen recording	14(21.9)	5(45.5)	3(15.0)	2(28.6)	4(17.4)
Scavenging system	15(23.4)	5(41.7)	4(22.2)	1(14.3)	5(21.7)
Breathing systems	44(68.8)	11(84.6)	10(50.0)	7(100)	16(69.6)
Operating table	61(95.3)	13(100)	18(85.7)	7(100)	23(100)
Suction machine	62(96.9)	13(100)	20(95.2)	7(100)	22(95.7)
Monitors					
Pulse oximeter	60(93.8)	13(100)	20(95.2)	7(100)	20(87.00)
Automated BP machine	55(85.9)	12(92.3)	18(85.7)	7(100)	18(78.3)
Manual BP machine	49(76.6)	9(69.2)	16(76.2)	3(42.9)	21(91.3)
ECG	23(35.9)	5(38.5)	7(33.3)	5(71.4)	6(27.3)
Temperature probe	14(21.9)	5(38.5)	2(10.0)	1(14.3)	6(26.1)
Capnograph	11(17.2)	5(38.5)	2(9.5)	3(42.9)	1(4.6)
Stethoscope	62(96.9)	13(100)	21(100)	7(100)	21(91.3)
Defibrillator	14(21.9)	4(30.8)	4(19.1)	4(57.1)	2(8.7)
Airway equipment					
Laryngoscope	62(96.9)	13(100)	21(100)	6(85.7)	22(95.7)
Face masks	63(98.4)	13(100)	21(100)	7(100)	22(95.7)
ETT and connections	60(93.8)	13(100)	19(95.0)	6(85.7)	22(95.7)
Artificial airways	59(92.2)	12(92.3)	18(85.7)	7(100)	22(95.7)
Ambu bag	63(98.4)	13(100)	21(100)	7(100)	22(95.7)
Magill’s forceps	50(78.1)	11(84.6)	17(85.0)	6(85.7)	16(69.6)
Difficult airway cart	4(6.3)	1(7.7)	1(5.0)	0	2(8.7)
Recovery room					
Recovery room present	28(43.8)	5(38.5)	3(14.3)	5(71.4)	15(65.2)
Pulse oximeter	10(15.6)	1(20.00)	1(33.3)	4(80.0)	4(26.7)
Automated BP machine	7(10.9)	0	0	3(75.0)	4(26.7)
Manual BP machine	5(7.8)	0	0	2(40.0)	3(20.0)
Temperature monitor	2(3.1)	0	0	1(20.0)	1(7.1)
ECG	3(4.7)	0	0	2(50.0)	1(7.1)
Continuous Pulse display	7(10.9)	0	0	3(75.0)	4(26.7)
Stethoscope	6(9.4)	0	0	3(60.0)	3(20.0)
Sanction machine	6(9.4)	0	0	3(60.0)	3(20.0)
Peripheral nerve stimulators	1(1.7)	1(7.7)	0	0	0
Oxygen supply	9(14.1)	1(20.00)	0	4(80.0)	4(26.7)

Table 3 shows the hospitals compliance to WFSA International standards for safe anaesthesia and only 3 facilities (4.7%) fulfilled the standards. In our study we considered 15 facility variables available in theatre and postoperative recovery areas including functional anaesthesia machine, oxygen source, reservoir oxygen source, continuous Blood Pressure, Continuous ECG, and Continuous pulse oximetry, suction machine, laryngoscope, Endotracheal tubes, Face Masks and Laryngeal Mask Airways, Stethoscope, Difficult Airway Cart, Defibrillator, Capnograph and availability of ICU facilities for post-operative care of complicated cases.

**Table 3:** Hospitals compliance to WFSA International standards for safe anaesthesia; Based on our description of WFSA international standards

Fulfill WFSA standards	Number of hospitals	Percentage (95%CI)
Yes	3^a^	4.69 (0–10.00)
No	61	95.31(89.99–100)

^a^Private hospitals

**Fig. 2:**
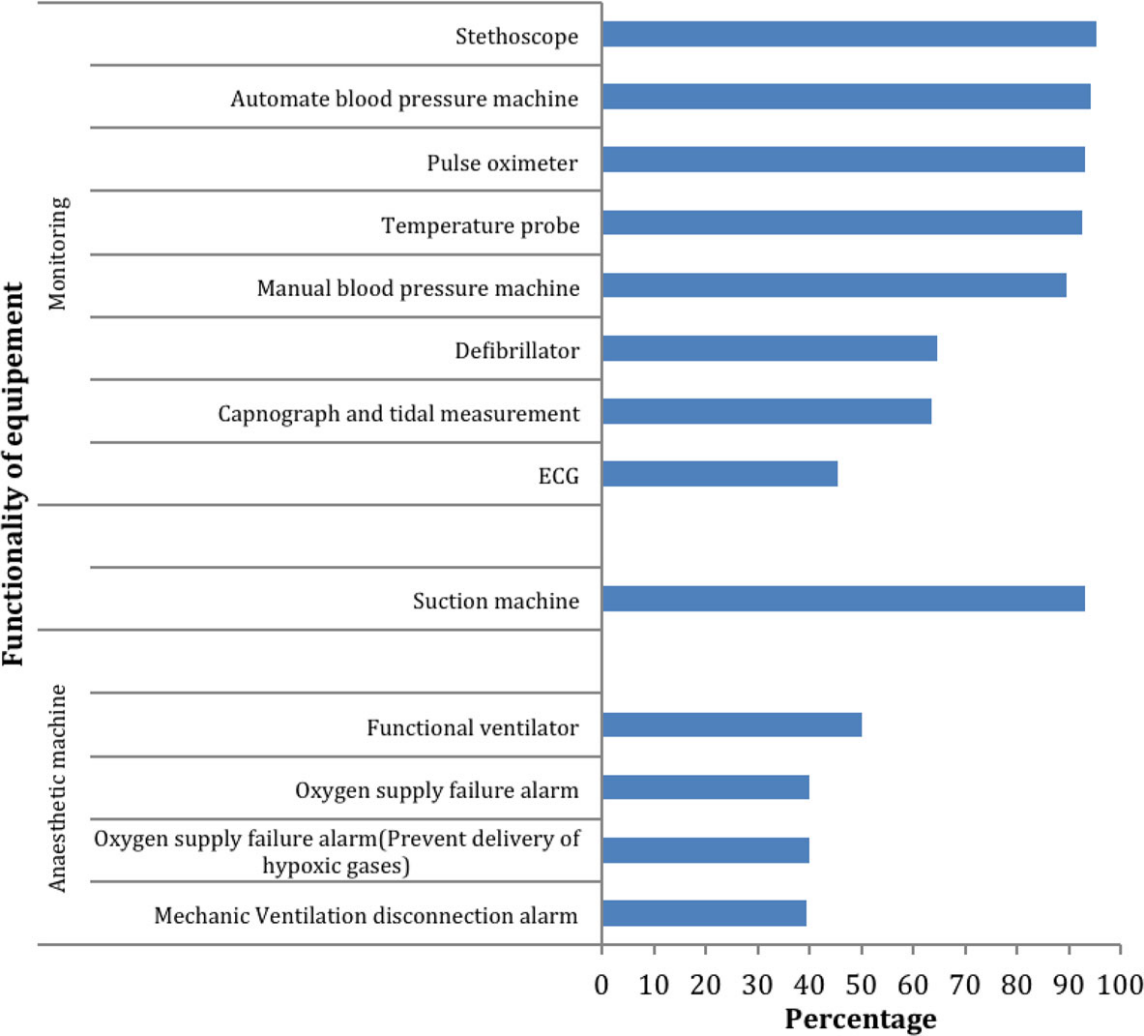
showing functionality of anaesthesia equipment in the hospitals

**Fig. 3:**
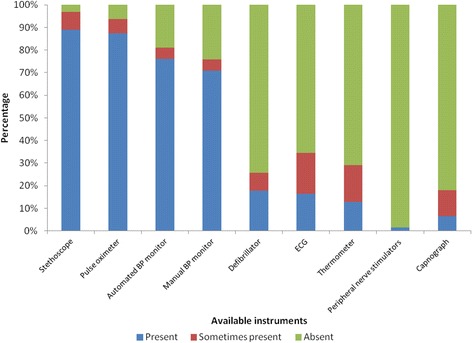
Availability and usage of monitors during cesarean sections at Hospitals in Uganda

We noted that many of the anaesthesia machines present were obsolete models without functional safety alarms and/or mechanical ventilators (Fig. 2). Our observations of postoperative recovery facilities revealed them to be poorly equipped in regional referral and general hospitals when compared to the private facilities (Table 2). Continuous ECG was only available in 3/64 (5%) of hospitals. Figure 3 shows the intraoperative availability and usage of monitoring equipment.

**Fig. 4:**
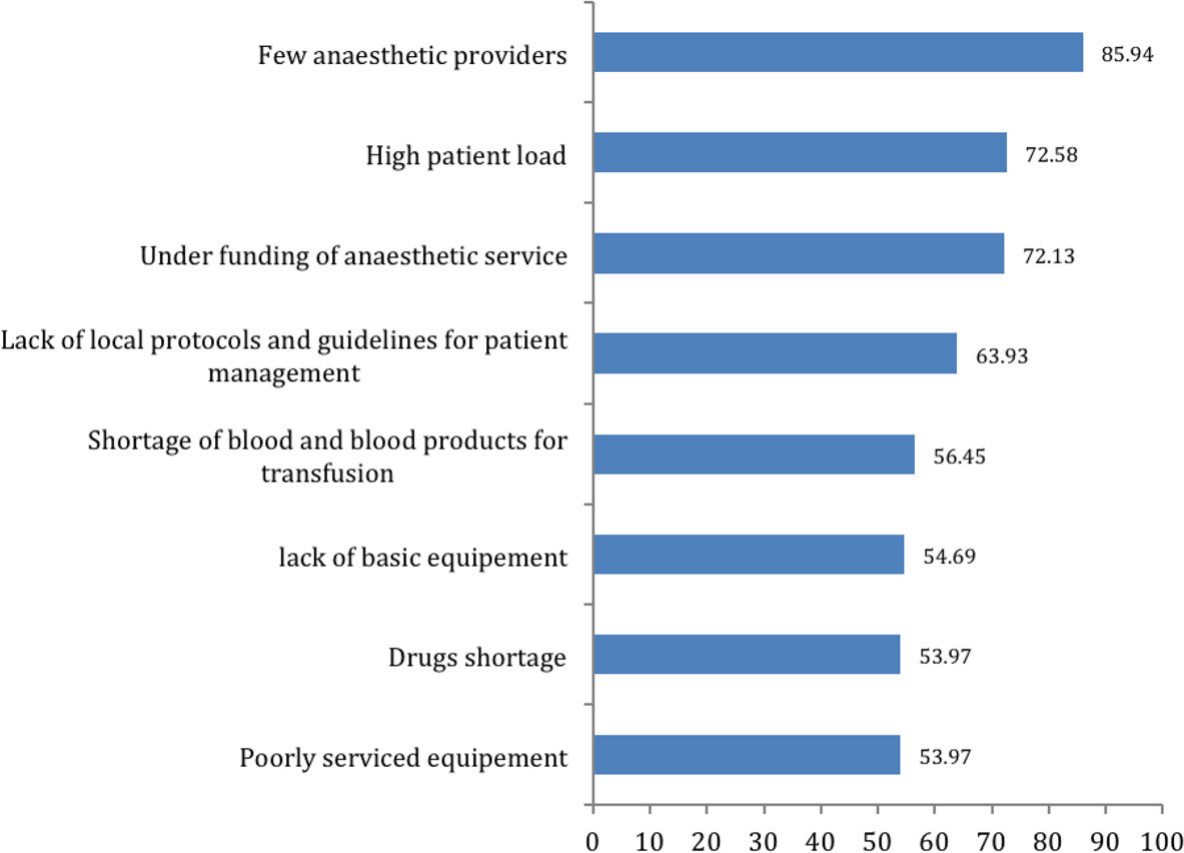
Showing the most common challenges faced in delivering obstetric anaesthesia in Uganda

The main challenges to provision of safe anaesthesia highlighted from the key informant interviews with the hospital directors at the study sites include: Few anaesthetic providers, a high patient load, under funding of the anaesthesia service and lack of local protocols to guide patient management (Fig. 4).

In 2015 there was a total of 79,417 cesarean sections reported from the Ministry of Health and 280 associate clinician anaesthetists as received from the District reports. For every 1000 cesarean sections there was an average of 4 associate clinician anaesthetists and 0.1 physician anaesthetist.

## Discussion

Our cross-sectional study represents almost 50% of all the hospitals in Uganda providing maternity and emergency surgery services. Of the 64 hospitals 4% did not have a trained physician anaesthetist, 92% had ≥1 nurse anaesthetist, while 8% had no trained anaesthesia staff at all; neither physician nor associate clinicians. In this 8% “anaesthetic assistants” who merely learnt a few limited techniques “on the job” conducted anaesthesia, or alternatively the district called in an anaesthetist from a neighbouring district to help and several patients were actually referred for surgery in other districts if they failed to get an anaesthetist.

In Uganda Anaesthesia is a growing discipline with an increase from 13 in 2007 [10] to just over 50 physician anaesthetists in the country in the last 10 years; mostly concentrated in larger urban areas. While there are about 280 associate clinician anaesthetists they are spread out thinly mostly in rural areas and yet these hospitals are barely equipped with the required facilities and drugs for safe anaesthesia. Complicated cases therefore need to be referred to bigger hospitals introducing significant delay in transport resulting in high maternal mortality and morbidity [11].

The global shortage of healthcare workers is estimated to be at 4.2 million, with approximately 1 million more workers needed in sub-Saharan Africa alone [12, 13]. In high-income countries fully accredited physician or nurse anesthetists provide anesthesia. Regrettably as we have identified in Uganda, there are parts of the world where anesthesia providers have only technical training, and sometimes not much of that. The WFSA views anesthesia as a medical practice, but acknowledges that access to anesthesia would not always be possible without the contribution of associate clinicians [14]. The mere availability of pulse oximetry will not, in and of itself, improve patient care. Anesthesia providers must actually make use of these monitors, and respond appropriately to the information they provide. This implies certain other resources, such as oxygen and the means to manage hypoventilation and maintain a patent airway. It also implies a fairly comprehensive knowledge of the relevant physiology, and an understanding of clinical anesthesia [14]. Safe anesthesia practice in any region depends on the knowledge and skills of those who live and work there. Education is the catalyst by which local standards can be improved, and it serves as the vehicle through which local aspirations to the internationally accepted standards of health care expected in the high-income world can be realized [14].

In a 2016 systematic review article [15], anaesthesia contributed to 13.8% of all maternal deaths after cesarean sections with the overall risk of any maternal death when associate clinicians provided care was 9.8 per 1000 (95% CI 5.2–15.7), compared to 5.2 per 1000 (0.9–12.6) for physician anaesthetists. The underlying causes were reported for 124 maternal deaths (24 studies). Of all deaths, 56 (45%) resulted from airway complications such as difficult or failed tracheal intubation, oesophageal intubation, bronchospasm, ventilation difficulties, and hypoxia; 38 (31%) from pulmonary aspiration; 34 (27%) from issues related to staff competency, poor pre-assessment, intraoperative monitoring, and equipment failure. Other causes included cardiac arrest at induction or during the procedure (seven [6%]), high spinal anaesthesia (eight [6%]), and drug overdose or adverse reactions (seven [6%]).

Aside from the limited number of anesthesia personnel in Uganda, there are frequent stock outs of regional anaesthetics and other essential drugs throughout the country. There was also variable availability of monitoring equipment in government and private hospitals. Only 3 (5%) of the hospitals had all the 15 variables in the operating theatre and recovery room, meeting the WFSA International standards for safe anaesthesia.

Other bottlenecks in provision of safe anaesthesia in Uganda included the lack of local protocols and guidelines for patient management, shortage of blood and blood products for transfusion, poorly serviced equipment and under funding of the anesthesia service.

The strengths of our study are that it was conducted at 64 hospitals in Uganda, representing 41% of all the hospitals in the country and 100% of the government regional referral hospitals, 15% of government district hospitals and 33% of all private hospitals in the country.

Our recommendations are hereby generalizable to low-income countries that face similar challenges. We acknowledge that this study is limited by the fact that it was a cross-sectional survey and some hospitals were purposefully selected for representation of government regional referral and district hospitals, and private for profit and not-for profit hospitals. This could have introduced a degree of selection bias, but individual anaesthetists were selected by simple random sampling depending on their availability at the hospital.

## Conclusions

While the United Nations Millennium Development Goals targeted a 75% reduction in maternal mortality ratio (MMR) by 2015, many countries failed to reach the goal and some failed to achieve any reduction at all. Uganda has still not met the target with a 2015 MMR of 343 deaths per 100,000 live births [16]. Many of these deaths are preventable and access to safe anaesthesia and quality cesarean section services have an important role.

We conclude that there is an urgent need to strengthen anaesthesia services in Uganda; to train more physician anaesthetists, improve training of associate clinicians and provide necessary equipment and drugs required for safe anaesthesia. These services are a key component of comprehensive emergency obstetric care and appropriately trained anaesthetists have a key role in the critically ill mother and ensuring improved surgical outcomes. In addition there is need to develop local guidelines for safe anaesthesia. This will be a great step towards implementing the World Health Assembly Resolution 68.15: *Strengthening Emergency and Essential Surgical Care as a Component of Universal Health Coverage*.

## Abbreviations

BP: Blood Pressure
ECG: Electrocardiogram
ETT: Endotracheal tube
ICU: Intensive care unit
LMA: Laryngeal mask airway
LMICs: Low-and middle-income countries
UCGHI: University of California Global Health Institute
UNCST: Uganda National Council for Science & Technology Ethics Committee
WFSA: World Federation of Societies’ of Anaesthesiologists
WHO: World Health Organization
